# Plant Derived Aporphinic Alkaloid S-(+)-Dicentrine Induces Antinociceptive Effect in Both Acute and Chronic Inflammatory Pain Models: Evidence for a Role of TRPA1 Channels

**DOI:** 10.1371/journal.pone.0067730

**Published:** 2013-07-04

**Authors:** Deise Prehs Montrucchio, Marina Machado Córdova, Adair Roberto Soares Santos

**Affiliations:** 1 Departamento de Farmácia, Setor de Ciências da Saúde, Universidade Federal do Paraná, Curitiba, PR, Brasil; 2 Programa de Pós-Graduação em Farmacologia, Universidade Federal de Santa Maria, Santa Maria, RS, Brasil; 3 Laboratório de Neurobiologia da Dor e Inflamação, Departamento de Ciências Fisiológicas, Universidade Federal de Santa Catarina, Florianópolis, SC, Brasil; University of Arizona, United States of America

## Abstract

S-(+)-Dicentrine is an aporphinic alkaloid found in several plant species, mainly from Lauraceae family, which showed significant antinociceptive activity in an acute model of visceral pain in mice. In this work, we extended the knowledge on the antinociceptive properties of S-(+)-dicentrine and showed that this alkaloid also attenuates mechanical and cold hypersensitivity associated with cutaneous inflammation induced by Complete Freund’s Adjuvant in mice. Given orally, S-(+)-dicentrine (100 mg/kg) reversed CFA-induced mechanical hypersensitivity, evaluated as the paw withdrawal threshold to von Frey hairs, and this effect lasted up to 2 hours. S-(+)-Dicentrine also reversed CFA-induced cold hypersensitivity, assessed as the responses to a drop of acetone in the injured paw, but did not reverse the heat hypersensitivity, evaluated as the latency time to paw withdrawal in the hot plate (50°C). Moreover, S-(+)-dicentrine (100 mg/kg, p.o.) was effective in inhibit nociceptive responses to intraplantar injections of cinnamaldehyde, a TRPA1 activator, but not the responses induced by capsaicin, a TRPV1 activator. When administered either by oral or intraplantar routes, S-(+)-dicentrine reduced the licking time (spontaneous nociception) and increased the latency time to paw withdrawal in the cold plate (cold hypersensitivity), both induced by the intraplantar injection of cinnamaldehyde. Taken together, our data adds information about antinociceptive properties of S-(+)-dicentrine in inflammatory conditions, reducing spontaneous nociception and attenuating mechanical and cold hypersensitivity, probably via a TRPA1-dependent mechanism. It also indicates that S-(+)-dicentrine might be potentially interesting in the development of new clinically relevant drugs for the management of persistent pain, especially under inflammatory conditions.

## Introduction

Pain is normally a transitory unpleasant sensation subsequent to a noxious or potentially injurious stimulus, acting as a warning system for tissue protection against injuries. It is a complex experience that involves not only the transduction of noxious environmental stimuli, but also cognitive and emotional processing by the brain [1], [2]. Some circumstances, such as inflammatory or neuropathic conditions, may lead to alterations of the pain pathway, leading to hypersensitivity, and the pain becomes chronic and debilitating. Indeed, hypersensitivity to heat, cold and mechanical stimuli are well documented symptoms of inflammatory and neuropathic pain [2], [3].

Several molecules and signaling pathways that contribute for noxious stimuli detection have already been characterized [1]. Among them, the transient receptor potential (TRP) ion channels appear to be molecular gateways in the sensory system [4]. In the field of pain, the subset of thermo-TRPs, mainly TRPV1 and TRPA1, seems to be important for initiation and maintenance of sensory nerve impulses that lead to nociception [5].

TRPA1 is a non-selective cation channel, expressed in primary sensory fibers that also express TRPV1. Around 97% of the TRPA1-expressing neurons also express TRPV1, while only 30% of fibers expressing TRPV1 also express TRPA1 [6], [7].

TRPA1 channels play a role in transduction of chemical and physical stimuli into electric nerve signals [8], being activated by irritant chemicals such as allylisothiocyanate from mustard oil, allicin from garlic, cinnamaldehyde from cinnamon and formalin [9], [10], [11], [12]. It is also a cold sensor, activated by temperatures below 17°C [7]. Inflammatory mediators such as bradykinin and prostaglandins can also indirectly activate TRPA1, thus, this channel is expected to be activated in inflammatory conditions [13]. Indeed, TRPA1 responses are increased in acute inflammatory process induced by Complete Freund’s Adjuvant (CFA) and this channel seems to be important in the maintenance of mechanical hypersensitivity [13], [14], [15], [16]. Thus, inflammatory sensitization of TRPA1 may underlie some components of inflammatory hypersensitivity, particularly to mechanical and cold stimuli [16], [17].

Several studies demonstrate that TRPA1 is involved in cold pain transduction, more specifically in pathophysiological cold hypersensitivity, since the use of TRPA1 antisense oligodeoxynucleotide reverses the cold hypersensitivity after CFA-induced inflammation [7], [18], [19].

The actual knowledge about TRPA1 channels points to a potential clinical use of TRPA1 antagonists for the control of pain states, however, the number of known selective TRPA1 inhibitors is surprisingly low [13], [17].

S-(+)-Dicentrine is an aporphinic alkaloid found in several plant species, mainly from Lauraceae family. Among its biological properties, it has been reported a vasodilator and antihypertensive action [20], [21], [22], platelet aggregation inhibition [23], [24] and even a cytostatic effect against some tumor cell lines from mice and humans [25], [26], [27], [28]. Recently, our research group reported that S-(+)-dicentrine has an important antinociceptive effect in a model of visceral pain in mice [29], which lead us to further investigate its effect on inflammatory models of pain, as well as possible mechanisms of action. In this context, here we investigate the antinociceptive effect of S-(+)-dicentrine in the CFA-induced inflammatory pain and show a possible involvement of TRPA1 in its mechanism of action.

## Methods

### Animals

Experiments were conducted using adult male Swiss mice (25–35 g) obtained from the animal facility of Universidade Federal de Santa Catarina (UFSC, Florianópolis, SC, Brazil) and housed in collective cages at 22±1°C under a 12-h light/dark cycle (lights on at 06∶00 h), with free access to food and water. Animals were habituated to the laboratory conditions for at least 1 h before testing and all experiments were performed during the light phase of the cycle. All animal care and experimental procedures were carried out in accordance with the National Institutes of Health Animal Care Guidelines (NIH publications No. 80-23), and conducted following the protocol approved by the Committee for the Ethical Use of Animals of the Universidade Federal de Santa Catarina (CEUA/UFSC, protocol number PP00462, approved in December 9^th^, 2010). All efforts were made to demonstrate consistent effects of the drug treatments and minimize the number of animals used and their suffering [30].

### Complete Freund’s Adjuvant-induced Chronic Inflammatory Pain

Mice received an intraplantar injection of 20 µL of Complete Freund’s adjuvant (CFA) (*Mycobacterium tuberculosis* heat killed and dried in paraffin oil), diluted at 50% or 80%, as described by Ferreira et al. [31] with minor modifications. Control group received 20 µL of vehicle (1% tween 80 in saline) intraplantar. CFA caused hind paw edema, mechanical and thermal hypersensitivity, evaluated 24 hours after CFA injection.

#### a) Measurement of mechanical hypersensitivity

Mechanical hypersensitivity was evaluated as previously described [32] with minor modifications. Mice were acclimatized in individual clear boxes (9×7×11 cm) on an elevated wire-mesh platform, to allow access to the ventral surface of the hindpaws, and mechanical hypersensitivity was assessed by the sensitivity to the application of von Frey hairs (Stoelting Co., Chicago, USA). The von Frey filaments (0.02–4.0 g) were presented perpendicularly to the plantar surface of the injected paw, and held in this position for 5 s with enough force to cause a slight bend in the filament. Positive responses included an abrupt withdrawal of the hindpaw or flinching behaviour immediately following removal of the stimulus. A median paw withdrawal threshold was determined using an adaptation of the Dixon’s up–down method [33].

#### b) Measurement of thermal (heat/cold) hypersensitivity

Thermal hypersensitivity to heat was evaluated as previously described by Eddy and Leimbach [34] and adapted by Luszczki and Czuczwar [35]. Animals were placed in a hot plate set at 50±1°C (Cold-hot Plate, AVS Projetos, Campinas, SP, Brazil) and the nociception was recorded as the latency time to withdrawal, shaking or licking the injected paw. A cut-off time of 20 s was used to avoid tissue damage. Cold hypersensitivity was evaluated as described by Flatters and Bennett [36] with minor modifications. Mice were placed in a wire mesh floor and a drop of acetone (20 µL) was gently sprayed in the ventral surface of the right hindpaw. Behavioral response was analyzed during 30 s and recorded in scores: 0– no response; 1– quick withdrawal, flick or stamp the paw; 2– prolonged withdrawal or repeated paw flicking; 3– repeated paw flicking with licking directed at the ventral side of the paw. Acetone application was repeated three times for each hindpaw, with intervals of five minutes, and the sum of the scores was used for data analysis.

### Capsaicin-induced Nociception

To evaluate the possible involvement of TRPV1 channels on S-(+)-dicentrine antinociceptive effect, mice were submitted to a test using capsaicin, a specific activator of these channels, as previously described by Santos et al. [37]. Mice were pretreated with vehicle (10 mL/kg, p.o.), S-(+)-dicentrine (100 mg/kg, p.o.) or AMG9810 (a selective TRPV1 antagonist used as positive control, 30 mg/kg, i.p.) 1 h (for p.o. administration) or 0.5 h (for i.p. administration) prior to the injection of 20 µL of capsaicin (TRPV1 activator, 1.6 µg/paw) in the plantar surface of the right hindpaw. Immediately after the capsaicin injection, animals were placed into clear observation chambers (9×11×13 cm) and the nociceptive response was evaluated as the time spent licking the injected paw during 5 min.

In another set of experiments, mice received vehicle (10 µL/paw) or S-(+)-dicentrine (100 µg/paw) by intraplantar route, co-injected with capsaicin (1.6 µg/paw), in a total volume of 20 µL. Immediately after the intraplantar injection, animals were placed into clear observation chambers (9×11×13 cm) and the nociceptive response was evaluated as the time spent licking the injected paw during 5 min.

### Cinnamaldehyde-induced Nociception

To evaluate the possible involvement of TRPA1 channels in S-(+)-dicentrine antinociceptive effect, mice were submitted to a test using cinnamaldehyde, a specific activator of these channels, as previously described by Cordova et al. [38]. Mice were pretreated with vehicle (10 mL/kg, p.o.), S-(+)-dicentrine (100 mg/kg, p.o.) or camphor (a TRPA1 antagonist used as positive control, 7.6 mg/kg, s.c.) 1 h (for p.o. administration) or 0.5 h (for s.c. administration) prior to the injection of 20 µL of cinnamaldehyde (TRPA1 activator, 1.3 µg/paw) in the plantar surface of the right hindpaw.

In another set of experiments, mice received vehicle (10 µL/paw) or S-(+)-dicentrine (100 µg/paw) by intraplantar route, co-injected with cinnamaldehyde (1.3 µg/paw), in a total volume of 20 µL. Immediately after the intraplantar injections, animals were placed into clear observation chambers (9×11×13 cm) and the nociceptive response was evaluated as the time spent licking the injected paw during 5 min.

Considering the results obtained with a single dose of S-(+)-dicentrine in this model, the next step was to evaluate the effects of increasing doses of S-(+)-dicentrine administered either by oral route (in order to evaluate a systemic effect) or intraplantar route (in order to evaluate a peripheral effect) in the licking time and in the hypersensitivity to cold. For this, mice were pretreated with increasing doses of S-(+)-dicentrine (10–100 mg/kg, p.o.) 1 h before the injection of 20 µL of cinnamaldehyde (1.3 µg/paw), or received a co-injection of S-(+)-dicentrine (10–100 µg/paw) with cinnamaldehyde (1.3 µg/paw), in a total volume of 20 µL. Immediately after the intraplantar injections, animals were placed into clear observation chambers (9×11×13 cm) and the time spent licking the injected paw was recorded for 5 min. Then, 10 min after cinnamaldehyde injection, the same animals were placed in a cold plate (Cold-hot Plate, AVS Projetos, Campinas, SP, Brazil) set at 5±1°C and the hypersensitivity was evaluated as the latency time to paw withdrawal. A cut-off time of 40s was used to avoid tissue damage.

### Drugs

The following substances were used: CFA, cinnamaldehyde and camphor (Sigma–Aldrich, St.Louis, MO), capsaicin and AMG9810 (Tocris Bioscience, Ellisville, Missouri, USA). S-(+)-Dicentrine was isolated from *Ocotea puberula* fruits in the Phytochemistry Laboratory from Pharmacy Department, Universidade Federal do Paraná, as previously described [29], with purity greater than 98%. All other chemicals were of analytical grade and obtained from standard commercial suppliers. S-(+)-Dicentrine was dissolved in 1% DMSO (Merck, Germany) and 5% Tween 80 (CRQ, Brazil) plus saline (NaCl 0.9%), CFA, AMG9810 and cinnamaldehyde were dissolved in 1% Tween 80 plus saline, and all other drugs were dissolved in saline. The final concentration of Tween 80 or DMSO did not exceed 5 and 1% respectively and did not cause any effect *per se*.

### Statistical Analysis

Results are presented as mean ± S.E.M. and the data were analyzed by one-way analysis of variance (ANOVA) followed by Student-Newman-Keuls post hoc test, except CFA-induced chronic inflammatory pain that was analyzed by two-way ANOVA followed by Bonferroni post hoc test. All statistical analyses were performed using GraphPad Prism 5.0 (GraphPad Software, San Diego, CA). *P* values less than 0.05 were considered significant.

## Results

### CFA-induced Mechanical Hypersensitivity

**Figure 1:**
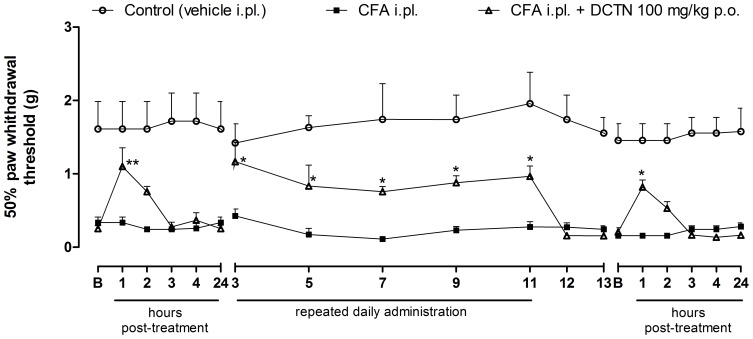
Effect of S-(+)-dicentrine (DCTN, 100 mg/kg, p.o.) on mechanical hypersensitivity induced by CFA 50%. On 1^st^ and 14^th^ days, evaluations were done 1, 2, 3, 4 and 24 hours post-DCTN treatment; all other evaluations were done 1 hour post-treatment. Each point represents the mean ± S.E.M. of 8 animals and significance levels are indicated by *p<0.05 and **p<0.01 when compared to the CFA i.pl. group (two-way anova and Bonferroni post hoc test).

**Figure 2:**
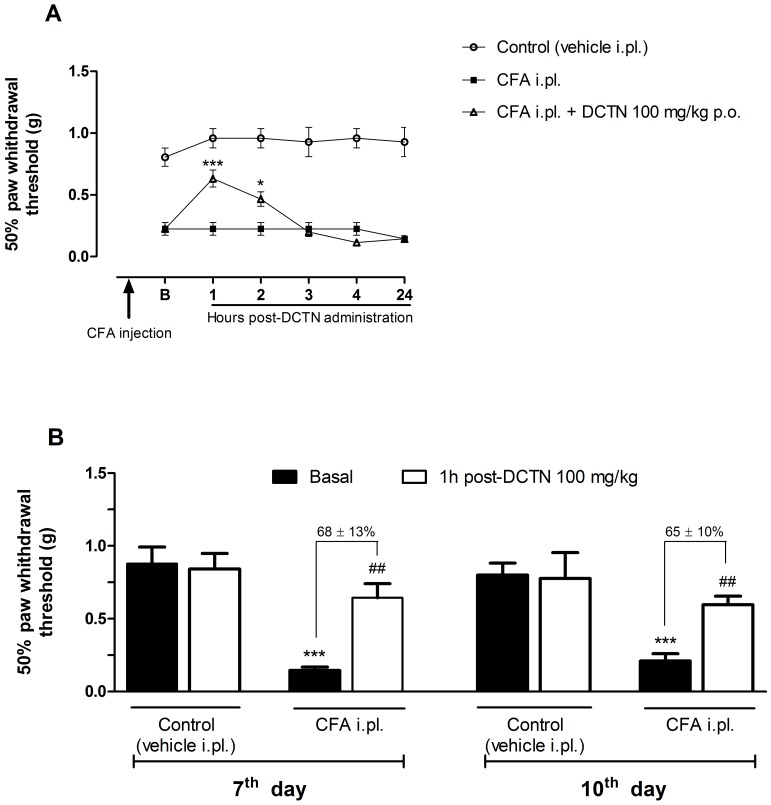
Effect of S-(+)-dicentrine (DCTN, 100 mg/kg, p.o.) on mechanical hypersensitivity induced by CFA 80%. Panel A: time-course effect of DCTN evaluated at 1, 2, 3, 4 and 24 hours post-DCTN administration; each point represents the mean ± S.E.M. of 10 animals and significance levels are indicated by *p<0.05 and ***p<0.001 when compared to the CFA i.pl. group (two-way anova and Bonferroni post hoc test). Panel B: effect of DCTN on 7^th^ and 10^th^ days post-CFA injection, evaluated before DCTN administration (basal) and 1 hour post-DCTN administration; each bar represents the mean ± S.E.M. of 10 animals and significance levels are indicated by ***p<0.001 when compared to control group and ^##^p<0.01 when compared to the respective basal of CFA i.pl. group (one-way anova and Student-Newman-Keuls post hoc test).

Considering the significant antinociceptive effect of S-(+)-dicentrine in acute models, found previously by our group [29], here we investigated whether S-(+)-dicentrine would be effective in a chronic inflammatory model of nociception. For this, mechanical hypersensitivity was evaluated 24 h after an intraplantar injection of CFA. As demonstrated in Fig. 1, CFA 50% caused mechanical hypersensitivity, which was characterized by the reduced paw withdrawal threshold when compared to the control group. S-(+)-Dicentrine (100 mg/kg, p.o.) was able to reverse mechanical hypersensitivity with a maximum effect 1 h post-treatment, and this antinociceptive effect was maintained while dicentrine was administered daily (100 mg/kg, p.o., once a day), until the 11^th^ day post-CFA injection. When treatment was interrupted for 2 days, mechanical hypersensitivity was re-established. On the 14^th^ day the treatment was restarted, and S-(+)-dicentrine was able to reduce mechanical hypersensitivity with a time-course effect profile similar to the first day post-CFA injection, indicating no tolerance effect. However, this concentration of CFA (50%) did not induce thermal hypersensitivity to cold (data not shown), which lead us to a second experiment using CFA at 80% of concentration. As shown in Fig. 2A, the time-course effect of S-(+)-dicentrine was similar to that obtained with CFA 50%, with an anti-hypersensitivity effect that lasted up to 2 h post-administration. Animals were treated daily with S-(+)-dicentrine and mechanical hypersensitivity was evaluated at the 7^th^ and 10^th^ days. Both groups (vehicle i.pl. and CFA i.pl.) were evaluated immediately before (basal) and 1 h post S-(+)-dicentrine administration. S-(+)-Dicentrine (100 mg/kg, p.o.) was able to reverse mechanical hypersensitivity with inhibitions of 68±13% and 65±10%, respectively, with no effect *per se* (Fig. 2B).

### CFA-induced Thermal Hypersensitivity

**Figure 3:**
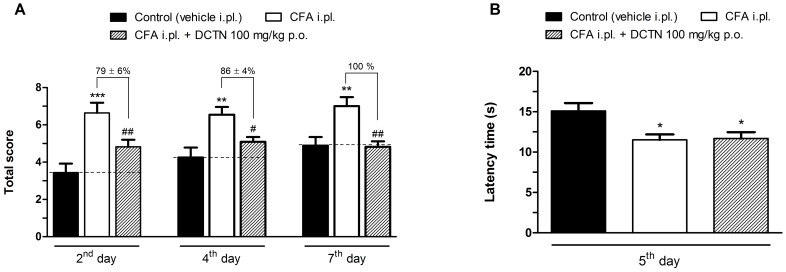
Effect of S-(+)-dicentrine (DCTN, 100 mg/kg, p.o.) on thermal hypersensitivity to cold (panel A) and heat (panel B), induced by CFA 80%. Each bar represents the mean ± S.E.M. of 10 animals. Significance levels are indicated by *p<0.05, **p<0.01 and ***p<0.001 when compared to control group and ^#^p<0.05 and ^##^p<0.01 when compared to the CFA i.pl. group (one-way anova and Student-Newman-Keuls post hoc test).

Hypersensitivity to cold stimulus was evaluated at 2^nd^, 4^th^ and 7^th^ day post-CFA injection and as showed in Fig. 3A, S-(+)-dicentrine (100 mg/kg, p.o.) was able to reduce the responses to acetone with inhibitions of 79±6%, 86±4% and 100% on 2^nd^, 4^th^, 7^th^ days, respectively. S-(+)-Dicentrine had no effect *per se* (data not shown). However, when evaluated in the hot-plate, dicentrine did not increase the latency time for paw withdrawal, indicating no effect on heat hypersensitivity (Fig. 3B).

### Capsaicin and Cinnamaldehyde-induced Nociception

**Figure 4:**
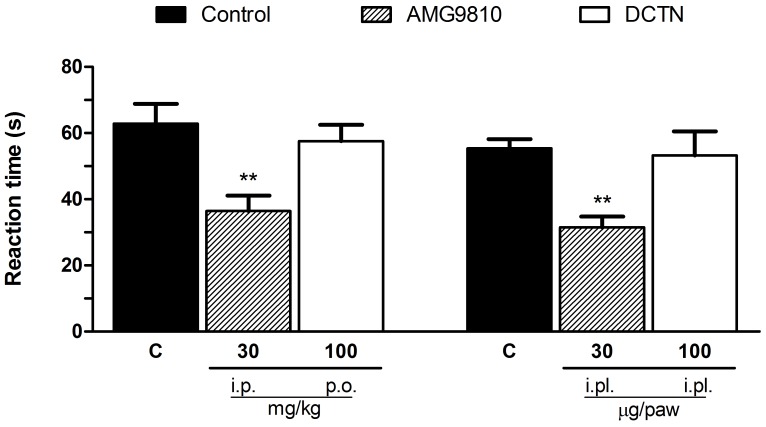
Effect of S-(+)-dicentrine (DCTN) administered by oral (100 mg/kg) or intraplantar (100 µg/paw) routes, or the TRPV1 antagonist AMG9810 by intraperitonial (30 mg/kg) or intraplantar (30 µg/paw) routes on capsaicin-induced nociception. Each bar represents the mean ± S.E.M. of 6 - 8 animals, being column C indicative of control values. Significance levels are indicated by **p<0.01 when compared to control group (one-way anova and Student-Newman-Keuls post hoc test).

**Figure 5:**
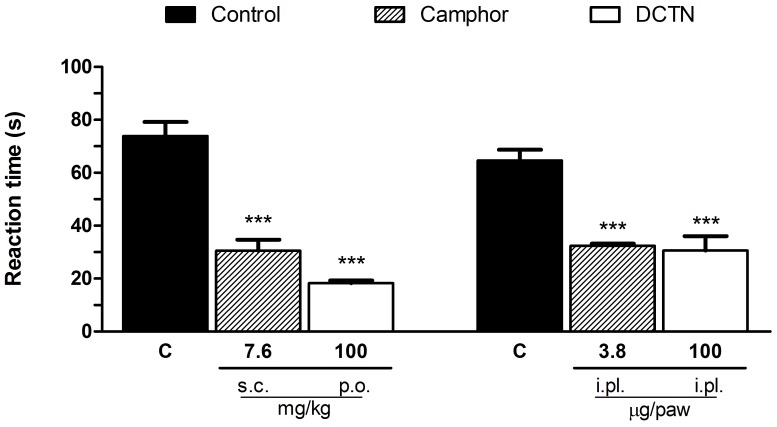
Effect of S-(+)-dicentrine (DCTN) administered by oral (100 mg/kg) or intraplantar (100 µg/paw) routes, or the TRPA1 antagonist camphor by subcutaneous (7.6 mg/kg) or intraplantar (3.8 µg/paw) routes on cinnamaldehyde-induced nociception. Each bar represents the mean ± S.E.M. of 6 - 8 animals, being column C indicative of control values. Significance levels are indicated by ***p<0.001 when compared to control group (one-way anova and Student-Newman-Keuls post hoc test).

Since S-(+)-dicentrine reduced hypersensitivity to cold, but not to heat, we further investigated if the thermo-TRPs (TRPV1 and TRPA1 ion channels) would be involved in on its effect. As showed in Fig. 4, the TRPV1 activator capsaicin induced a licking behavior characteristic of nociception, which was reduced by the TRPV1 antagonist AMG9810, either when administered by intraperitoneal or intraplantar routes, but not by S-(+)-dicentrine. However, as showed in Fig. 5, S-(+)-dicentrine was able to reduce the licking time induced by cinnamaldehyde, a TRPA1 activator, either when administered by oral route (100 mg/kg) or intraplantar (100 µg/paw), with inhibitions of 75±1% and 53±8%, respectively, similarly to the TRPA1 blocker camphor.

**Figure 6:**
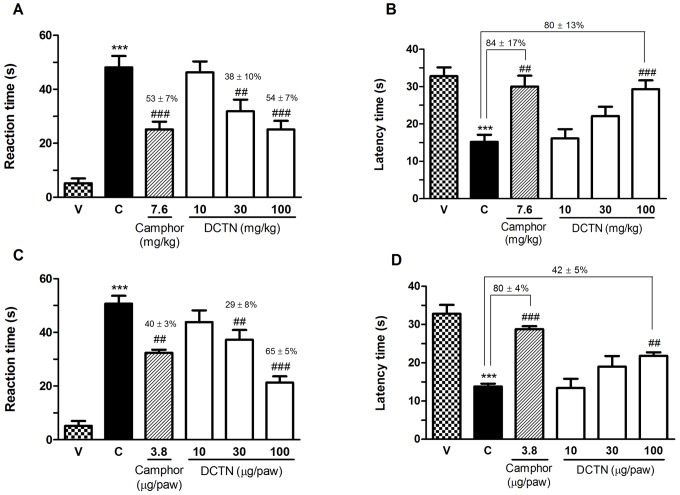
Effect of S-(+)-dicentrine (DCTN) administered by oral route (10 - 100 mg/kg, panels A and B) or by intraplantar route (10 - 100 µg/paw, panels C and D), or the TRPA1 antagonist camphor by subcutaneous (7.6 mg/kg) or intraplantar (3.8 µg/paw) routes on cinnamaldehyde-induced nociception. Panels A and C represents the spontaneous nociception (licking time) and panels B and D represents the hypersensitivity to cold (latency time to paw withdrawal). Each bar represents the mean ± S.E.M. of 6 - 8 animals, being column C indicative of control values (cinnamaldehyde i.pl. injection) and column V indicative of group receiving only vehicle i.pl. injection. Significance levels are indicated by ***p<0.001 when compared to vehicle (V) group and ^##^p<0.01 and ^###^p<0.001 when compared to respective control (C) groups, and the values above the symbols represent the percent of inhibition (one-way anova and Student-Newman-Keuls post hoc test).

Given the indicative participation of TRPA1 on S-(+)-dicentrine effect, a dose-response curve was made evaluating both spontaneous nociception and cold hypersensitivity. As demonstrated on Fig. 6, S-(+)-dicentrine was able to reduce the licking time and also increase the latency time on the cold plate, both in a dose-related manner. When given by oral route (Fig. 6 A and B), S-(+)-dicentrine (30 and 100 mg/kg) produced an inhibition of spontaneous nociceptive response (licking) with inhibitions of 38±10% and 54±7%, respectively, similar to the inhibition of 53±7% of the positive control camphor. In the cold plate, S-(+)-dicentrine (100 mg/kg) increased the latency time for paw withdrawal in 80±13%, similar to the positive control camphor (84±17%). When administered by intraplantar route, co-injected with cinnamaldehyde, S-(+)-dicentrine (30 and 100 µg/paw) also produced an inhibition of licking time with inhibitions of 29±8% and 65±5%, respectively, while the positive control camphor produced an inhibition of 40±3%. In the cold plate, the dose of 100 µg/paw increased the latency time in 42±5%, while the positive control camphor increased the latency time in 80±4% (Fig. 6 C and D).

## Discussion

The nociceptive response begins when primary sensory fibers are activated by some noxious stimulus, which may be chemical, thermal or mechanical. The TRP ion channels, especially TRPV1 and TRPA1, are highly involved in the transduction and sensitization in primary afferent somatosensory neurons. Besides activated by irritant chemicals, these ion channels are transducers of both thermal and mechanical stimuli, acting as molecular integrators for a range of diverse noxious stimuli [3], [39]. Both TRPV1 and TRPA1 play an integral role in pain and neurogenic inflammation via sensory nerve activation, either at central or peripheral level [40]. Thus, the development of blockers of these ion channels may be of clinical interest for the control of chronic pain states.

Previous results from our research group have shown that a chloroform fraction obtained from an extract of *O. puberula* fruits, when given orally, was able to reduce mice nociceptive behavior induced by acetic acid, and we then demonstrated that this antinociceptive effect was partly related to the presence of S-(+)-dicentrine [29]. In the present work, we extended the knowledge on the antinociceptive effects of S-(+)-dicentrine using a chronic inflammatory model, and point to a possible interaction of this alkaloid with TRPA1 ion channels.

TRPA1 is expressed in sensory neurons of dorsal root ganglion (DRG), nodose ganglion (NG) and trigeminal ganglion neurons (TG) [7] and its role in peripheral detection of a variety of noxious stimuli is well established [41]. Peripheral application of TRPA1 agonists produces excitation of small diameter afferent fibers, leading to pain and hyperalgesia, which are reversed by peripheral application of TRPA1 antagonists [13], [41]. However, less is known about the role of TRPA1 channels on spinal nociceptive transmission [41], [42]. TRPA1 channels are expressed not only on distal, but also on central endings of primary afferent nociceptive fibers that are located within the spinal dorsal horn [8], [42]. On central endings, activation of TRPA1 is thought to facilitate glutamate release, enhancing frequency and amplitude of glutamatergic transmission of the afferent signal to spinal dorsal horn neurons [8], [42]. On the same line, Uta et al [43] demonstrated that the activation of spinal TRPA1 by cinnamaldehyde enhances the excitatory synaptic transmission.

TRPA1 channels can also be activated/modulated by endogenous agonists, such as oxidative stress products (hydrogen peroxide and 4-hydroxynonenal, for instance), nitric oxide, bradykinin, PAR-2 agonists and reactive prostaglandins such as 15d-PGJ2, produced following an initial inflammatory sign [8], [40], [44], [45], [46]. Some of these endogenous TRPA1 agonists are generated and appear in increased levels on painful conditions, like inflammatory processes. Thus, TRPA1 in nerve endings becomes over-activated by these inflammatory mediators and greatly contributes towards hypersensitivity associated with chronic pain states [8], [44].

In this work we used a model of peripheral inflammation induced by CFA, which mimics a chronic inflammatory condition, and we showed that S-(+)-dicentrine was able to reduce mice nociceptive responses of mechanical and cold hypersensitivity, but not those of heat hypersensitivity. It is well established that under inflammatory conditions, TRPV1 and TRPA1 are some of the main transducers of nociceptive response [3]. Since inflammation is usually associated with tissue acidosis, TRPV1 channels may be directly activated by protons, leading to the nociceptive transmission, besides being involved in the hypersensitivity to heat, commonly associated with chronic inflammation [47]. TRPA1 channels, besides mediate cold hypersensitivity associated with inflammatory conditions [39], also have their role in the transduction of mechanical stimuli extensively reported, although the exact mechanism by which they are involved in pain transmission is still not clear [3], [15], [48], [49]. In inflammatory models of nociception, such as formalin and CFA, TRPA1 channels seem to play a major role since pharmacological or genetic blockade of these channels substantially attenuate pain-related responses to formalin [12], [39] and consistently prevent the initial development and the maintenance of mechanical hyperalgesia following CFA injection in mice [13]–[16].

Regarding thermo sensation, TRPV1 and TRPA1 channels are the main transducers of noxious heat and cold, respectively, responding to temperatures above 43°C or below 17°C [7], [50], and sensitization of these channels have been reported as crucial for thermal hyperalgesia in pathological conditions [51]. Indeed, some reports have shown that treatment with TRPA1 antisense oligodeoxynucleotides reduces behavioral hypersensitivity to cold after CFA-induced inflammation or sciatic nerve injury, and TRPA1 knockout mice exhibits impaired behavioral responses to a cold plate maintained at 0°C [19], [45]. Furthermore, cold stimulus was found to potentiate the activation of TRPA1 caused by allylisothiocyanate and 4-hidroxynonenal, which is consistent with the assumption of cold hypersensitivity being driven by TRPA1 under inflammatory conditions [18], [44]. However, although several reports point to TRPA1 as important channels for cold sensation [7], [9], [13], [19], [44], [45], [48], their role on cold nociception is still controversial, since there are several other studies showing no impairment in cold sensation on TRPA1 deficient mice [6], [11], suggesting the presence of other sensors for colder temperatures, in addition to TRPA1. Indeed, the transient receptor potential melastatin 8 (TRPM8) has also been proposed to act as this cold sensor. Recent studies have demonstrated that TRPM8 is important for the detection of both cooling sensation and noxious cold, since TRPM8 knockout mice have impaired behavioral responses in models such as sensitivity to acetone and cold plate in inflammatory and neuropathic conditions [52]–[55].

Taking this into account, it is reasonable to think that the antihypersensitivity effect of S-(+)-dicentrine in the CFA model may be mediated by TRPs. To test this hypothesis, and considering that TRPV1 and TRPA1 are highly involved in the CFA-induced mechanical hypersensitivity, we evaluated the antinociceptive effect of S-(+)-dicentrine against specific activators of these two channels. Capsaicin, a selective activator of TRPV1, induced a nociceptive behavior that was reversed by AMG9810, a selective blocker of TRPV1, but not by S-(+)-dicentrine. This finding is in line with the results on CFA model, when dicentrine did not reverse the heat hypersensitivity, suggesting that S-(+)-dicentrine do not interact with TRPV1 channels. On the other hand, when cinnamaldehyde (a selective activator of TRPA1) was used, S-(+)-dicentrine was able to reverse the licking time indicative of nociception and also increase the latency time of paw withdrawal in the cold plate, indicative of cold hypersensitivity. These findings are in line with the results on CFA model, when S-(+)-dicentrine reduced both mechanical and cold hypersensitivity. Besides, Lennertz et al. [16] reported that CFA-induced inflammation increased the responses to mechanical stimuli in a subset of C fibers that are sensitive to both mechanical and cold stimuli, but not in the heat-sensitive C fibers, indicating that TRPA1 (but not TRPV1) contribute to mechanical sensitization in the CFA model. Taking this into account, our results strongly suggest that S-(+)-dicentrine acts through interaction with TRPA1 channels. However, considering the controversial data about the roles of TRPA1 and TRPM8 on cold hypersensitivity, a possible interaction of S-(+)-dicentrine with TRPM8 channels cannot be discarded. Thus, it would be interesting to further investigate the possible role of TRPM8 in the antinociceptive mechanism of action of S-(+)-dicentrine.

Considering the actual knowledge about the indicative participation of TRPs, specially TRPA1, in modulation of painful conditions associated with inflammatory and neuropathic pain states, these channels constitute an interesting target for the development of new analgesic drugs [13], [41]. The results presented here clearly point to an interaction with TRPA1 channels as a possible mechanism of action of S-(+)-dicentrine. If this is a direct or indirect interaction, through other intracellular signaling pathways, remains to be elucidated. Our results suggest that dicentrine may be an interesting molecule for further investigations on nociception, thus, other possible mechanisms for the S-(+)-dicentrine effect should be considered for further investigations.

### Conclusion

S-(+)-Dicentrine has an important antinociceptive effect in inflammatory conditions, reducing spontaneous nociception and attenuating mechanical and cold hypersensitivity associated with these conditions. This effect appears to be due to an interaction of S-(+)-dicentrine with TRPA1 channels, although the exact mechanism of this interaction is not clear. Taken together, our data adds information about antinociceptive properties of S-(+)-dicentrine and also indicates that it might be potentially interesting in the development of new clinically relevant drugs for the management of persistent pain, especially under inflammatory conditions.
